# Association of Nutritional Support With Clinical Outcomes Among Medical Inpatients Who Are Malnourished or at Nutritional Risk: An Updated Systematic Review and Meta-analysis

**DOI:** 10.1001/jamanetworkopen.2019.15138

**Published:** 2019-11-20

**Authors:** Filomena Gomes, Annic Baumgartner, Lisa Bounoure, Martina Bally, Nicolaas E. Deutz, Jeffrey L. Greenwald, Zeno Stanga, Beat Mueller, Philipp Schuetz

**Affiliations:** 1 University Department of Medicine, Clinic for Endocrinology, Diabetology, and Metabolism, Kantonsspital Aarau, Aarau, Switzerland and Medical Faculty of the University of Basel, Basel, Switzerland; 2 Nutrition Science Group, The New York Academy of Sciences, New York; 3 Center for Translational Research in Aging and Longevity, Department of Health & Kinesiology, Texas A&M University, College Station; 4 Core Educator Faculty, Department of Medicine, Massachusetts General Hospital, Boston; 5 Division of Diabetology, Endocrinology, Nutritional Medicine, & Metabolism, University Hospital Inselspital Bern, University of Bern, Bern, Switzerland

## Abstract

**Importance:**

Malnutrition affects a considerable proportion of the medical inpatient population. There is uncertainty regarding whether use of nutritional support during hospitalization in these patients positively alters their clinical outcomes.

**Objective:**

To assess the association of nutritional support with clinical outcomes in medical inpatients who are malnourished or at nutritional risk.

**Data Sources:**

For this updated systematic review and meta-analysis, a search of the Cochrane Library, MEDLINE, and Embase was conducted from January 1, 2015, to April 30, 2019; the included studies were published between 1982 and 2019.

**Study Selection:**

A prespecified Cochrane protocol was followed to identify trials comparing oral and enteral nutritional support interventions with usual care and the association of these treatments with clinical outcomes in non–critically ill medical inpatients who were malnourished.

**Data Extraction and Synthesis:**

Two reviewers independently extracted data and assessed risk of bias; data were pooled using a random-effects model.

**Main Outcomes and Measures:**

The primary outcome was mortality. The secondary outcomes included nonelective hospital readmissions, length of hospital stay, infections, functional outcome, daily caloric and protein intake, and weight change.

**Results:**

A total of 27 trials (n = 6803 patients) were included, of which 5 (n = 3067 patients) were published between 2015 and 2019. Patients receiving nutritional support compared with patients in the control group had significantly lower rates of mortality (230 of 2758 [8.3%] vs 307 of 2787 [11.0%]; odds ratio [OR], 0.73; 95% CI, 0.56-0.97). A sensitivity analysis suggested a more pronounced reduction in the risk of mortality in recent trials (2015 or later) (OR, 0.47; 95% CI, 0.28-0.79) compared with that in older studies (OR, 0.94; 95% CI, 0.72-1.22), in patients with established malnutrition (OR, 0.52; 95% CI, 0.34-0.80) compared with that in patients at nutritional risk (OR, 0.85; 95% CI, 0.62-1.18), and in trials with high protocol adherence (OR, 0.67; 95% CI, 0.54-0.84) compared with that in trials with low protocol adherence (OR, 0.88; 95% CI, 0.44-1.76). Nutritional support was also associated with a reduction in nonelective hospital readmissions (14.7% vs 18.0%; risk ratio, 0.76; 95% CI, 0.60-0.96), higher energy intake (mean difference, 365 kcal; 95% CI, 272-458 kcal) and protein intake (mean difference, 17.7 g; 95% CI, 12.1-23.3 g), and weight increase (0.73 kg; 95% CI, 0.32-1.13 kg). No significant differences were observed in rates of infections (OR, 0.86; 95% CI, 0.64-1.16), functional outcome (mean difference, 0.32; 95% CI, −0.51 to 1.15), and length of hospital stay (mean difference, −0.24; 95% CI, −0.58 to 0.09).

**Conclusions and Relevance:**

This study’s findings suggest that despite heterogeneity and varying methodological quality among trials, nutritional support was associated with improved survival and nonelective hospital readmission rates among medical inpatients who were malnourished and should therefore be considered when treating this population.

## Key Points

**Question:**

What is the association of nutritional support with clinical outcomes in medical inpatients who are malnourished or at nutritional risk?

**Findings:**

In this updated systematic review and meta-analysis of 27 trials including 6803 patients, nutritional support provided during hospitalization was associated with significantly lower rates of mortality and nonelective hospital readmissions, as well as higher energy and protein intake and weight increase.

**Meaning:**

This study’s findings suggest that nutritional support in hospitalized patients who are malnourished or at nutritional risk is associated with improved nutritional and clinical outcomes and should be considered when treating this population.

## Teaser

This systematic review and meta-analysis assesses whether nutritional support is associated with better clinical outcomes in medical inpatients who are malnourished or at nutritional risk.

## Introduction

Malnutrition is a major public health problem, particularly in the multimorbid medical population, affecting more than 30% of hospitalized patients.^1,2,3,4^ It results from the complex interplay of different predisposing factors, including immobilization and advanced age and the associations of illness with protein and energy homeostasis, protein catabolism, hormonal function, and appetite that lead to progressive weight loss and sarcopenia.^5,6^

Malnutrition is a major risk factor associated with high mortality and morbidity, functional decline, prolonged hospital stays, and increased health care costs.^2,7^ Nutritional support, when provided during the hospital stay, may offset some of these adverse outcomes. For this reason, international societies^4,8^ recommend screening patients for malnutrition risk and using nutritional support in patients at nutritional risk or who are malnourished. However, these recommendations have been largely based on physiological rationales. Two meta-analyses of trials investigating the use of nutritional support for medical and mixed medical, surgical, and critically ill inpatients did not find significant associations with outcomes, including mortality and several complications.^9,10^ Yet, the quality of the included studies was low, limiting any strong conclusions.

Considering these results, some authors have argued against the routine use of nutritional support in treating medical inpatients at nutritional risk and classified nutritional interventions as “services for which harms are likely to outweigh benefits.”^11^ Since the publication of the previously mentioned meta-analyses,^9,10^ however, several large, high-quality trials were published that may change the overall conclusions. Therefore, our aim was to perform an updated systematic review and meta-analysis to assess the associations of nutritional support with clinical outcomes in non–critically ill medical inpatients with malnutrition or at nutritional risk, overall and stratified by different subgroups.

## Methods

The methods used for this updated systematic review and meta-analysis were consistent with an initial analysis,^9^ which followed a prespecified Cochrane protocol^12^ and the Preferred Reporting Items for Systematic Reviews and Meta-analyses (PRISMA) reporting guidelines,^13^ as summarized below.

### Data Sources and Searches

The literature searches were conducted in the Cochrane Library, MEDLINE, and Embase electronic databases from January 1, 2015, just after the last date reviewed in the prior meta-analysis,^9^ to April 30, 2019. An example of the search strategy used in MEDLINE is provided in the eAppendix in the Supplement. In addition, we searched bibliographies of review articles and the ClinicalTrials.gov registry for ongoing or unpublished trials. Authors of ongoing nutritional support studies were also contacted. There were no language restrictions.

### Study Selection

We systematically searched the literature to identify randomized and nonrandomized clinical trials (RCTs) that allocated non–critically ill medical inpatients who are malnourished or at nutritional risk (based on body mass index, the presence of a disease associated with malnutrition, or the use of a nutritional assessment or screening tool) to a nutritional support intervention or a control group. Medical inpatients were defined as patients hospitalized in medical wards of acute care institutions (including those of geriatrics, gastroenterology, cardiology, pulmonology, general internal medicine, infectious diseases, nephrology, and oncology). The exclusion criteria were as follows: studies conducted in outpatient care settings, nursing homes, long-term care facilities, or intensive care units and trials focusing on surgical patients, patients with pancreatitis (because of their particular nutritional needs and the management of this condition), and those receiving palliative care.

We included studies with interventions consisting of any type of nutritional support (including dietary advice, changes in the organization of nutritional care, food fortification, extra snacks, oral nutrition supplements, and enteral tube feeding) except parenteral nutrition, independent of the duration of the intervention.

The primary study outcome was all-cause mortality, defined as death from any cause and measured at hospital discharge or at follow-up (up to 6 months after randomization). Secondary end points included nosocomial infections, nonelective readmissions, functional outcome (assessed by the Barthel index score at follow-up), length of hospital stay (LOS), daily energy and protein intake, and body weight change. We also gathered information about adherence to the nutritional intervention and the study protocol. Older studies were defined as those published before 2015^14,15,16,17,18,19,20,21,22,23,24,25,26,27,28,29,30,31,32,33^ (included in the original meta-analysis^9^) and newer studies as those published since 2015^34,35,36,37,38^ (identified in the updated meta-analysis).

### Data Extraction and Quality Assessment

Two of us (F.G. and A.B.) independently screened abstracts, extracted relevant data from the studies that met the inclusion criteria, and assessed their risk of bias. Disagreements were resolved by consulting one of us (P.S.). Two of us (A.B. and L.B.) assessed the trials in which another 2 of us (N.E.D. and P.S.) were directly involved.^36,38^ As recommended by the Cochrane Collaboration, the following criteria were used to assess risk of bias: random sequence generation (selection bias); randomization concealment (selection bias); blinding (performance bias and detection bias), separated for blinding of participants and personnel, and blinding of outcome assessment; incomplete outcome data (attrition bias); selective reporting (reporting bias); and other bias.

### Statistical Analysis

Dichotomous data were reported as odds ratios (ORs) or risk ratios (RRs) with 95% CIs and continuous data as the mean differences with 95% CIs. Data were pooled using a random-effects model.

We identified heterogeneity through visual inspection of the forest plots and also considered the *I*^2^ statistic, which quantifies inconsistency across studies. An *I*^2^ statistic value of 50% or more indicates a considerable level of heterogeneity. We used visual inspection of funnel plots to assess publication bias.

We conducted the following subgroup analyses: stratification by degree of malnutrition (established malnutrition vs risk of malnutrition), by baseline mortality rate in the control group (high mortality [≥10%] vs low mortality [<10%]), by adherence to the nutrition protocol (high adherence vs low adherence, as described in the eTable in the Supplement), by route of nutritional support (oral vs mixed routes), and by publication year (older [2014 or earlier] vs newer [2015 or later]).

All of the analyses were conducted with statistical significance set at *P* = .05, and the testing was 2-sided. Most figures were produced using Review Manager, version 5.3 (Cochrane Collaboration).

## Results

After discarding duplicates, we identified 265 abstracts from the 3 electronic databases and 5 additional records through manual searches and contact with experts. Five new eligible trials including 3067 participants that were published between 2015 and 2019 were identified. Among these 5 trials were 2 large trials including 652 patients^36^ and 2028 patients.^38^ Data from these 5 new trials were extracted and added to the original data file.^9^ The final analysis included a total of 27 trials with 6803 patients (including 5 from the new search and 22 from the previous one) (eFigure 1 in the Supplement). Table 1 provides an overview of the characteristics of these included studies.

**Table 1.** Overview of Included Studies

Source	Patient Population	Country	Total Sample Size	Intervention Group	Control Group
Bonilla-Palomas et al,^34^ 2016	Acute decompensated heart failure	Spain	120	Conventional treatment for heart failure combined with an individualized nutritional intervention: diet optimization, specific recommendations, ONS if nutritional goals were not reached, for 6 mo	Conventional treatment for heart failure
Broqvist et al,^14^ 1994	Acute decompensated heart failure	Sweden	21	Normal hospital food and between meals with 500 mL ONS daily containing 30 g protein and 750 kcal	Normal hospital food and 1:10 diluted placebo version of ONS
Bunout et al,^15^ 1989	Alcoholic liver disease	Chile	36	Oral diet including 50 kcal/kg/d, 1.5 g protein/kg/d, casein-based product	Standard diet
Cano-Torres et al,^35^ 2017	General medical inpatients	Mexico	55	Individualized nutrition plan according to energy and protein (1.0-1.5 g/kg) intake requirements as well as dietary advice based on face-to-face interviews with patients and their caregivers or family members, until hospital discharge	Standard nutritional management
Deutz et al,^36^ 2016	General medical inpatients (≥65 y of age)	United States	652	2 Bottles ONS daily providing 700 kcal/d, 40 g protein/d, 3 g calcium- beta-hydroxybeta-methylbutyrate, 160 IU vitamin D, and other essential micronutrients, for 90 d	2 Bottles placebo ONS providing 96 kcal and 20 mg vitamin C
Feldblum et al,^33^ 2011	General medical inpatients (≥65 y of age)	Israel	259	Individual nutritional treatment, 237 mL containing 12.6 g fat, 13 g protein, and 47.3 g carbohydrates (total, 360 kcal), additional food fortification	Routine care on request
Gariballa et al,^16^ 2006	General medical inpatients (≥65 y of age)	United Kingdom	445	2 Bottles (200 mL each) ONS daily, 995 kcal/d plus vitamins	Oral placebo (60 kcal)
Gazzotti et al,^17^ 2003	General medical inpatients (≥75 y of age)	Belgium	80	Standard hospital food and 1 Clinutren soup, 500 kcal/d, 21 g protein/d	Standard hospital food, no supplements
Hickson et al,^18^ 2004	General medical inpatients (≥65 y of age)	United Kingdom	592	Nutritional care from health care assistants, snacks and drinks	Usual care
Hogarth et al,^19^ 1996	General geriatric inpatients	United Kingdom	25	Intervention 1: daily 750 mL oral glucose supplement (540 kcal) and capsules containing vitamins A (8000 U), B_1_ (15 mg), B_2_ (15 mg), B_3_ (50 mg), B_6_ (10 mg), and C (500 mg), for 1 mo Intervention 2: daily 750 mL oral glucose supplement (540 kcal) and placebo capsules for 1 mo	Control 1: Nutrasweet glucose drink and capsules containing vitamins A (8000 U), B_1_ (15 mg), B_2_ (15 mg), B_3_ (50 mg), B_6_ (10 mg), and C (500 mg), for 1 mo Control 2: Nutrasweet glucose drink and placebo capsules for 1 mo
Holyday et al,^20^ 2012	General geriatric inpatients	Australia	143	Individual modification of hospital meals (fortification), nutrition supplements	Individual modification only on request
Huynh et al,^37^ 2015	General medical inpatients	India	212	Dietary counseling +2 bottles ONS daily providing 432 kcal/d and 16 g protein/d plus micronutrients, for 12 weeks	Dietary counseling alone
McEvoy and James,^21^ 1982	General medical inpatients	United Kingdom	54	2 Sachets oral “Build-Up” daily, 36.4 g protein and 644 kcal	Normal hospital diet
McWhirter and Pennington,^22^ 1996	General medical inpatients	United Kingdom	86	(a) ONS containing 566 kcal/d, 23.9 g protein/d (b) Nocturnal tube feeding (nasogastric tube), additional intake of 84 kcal/d and 29.5 g protein/d	Standard hospital diet
Munk et al,^23^ 2014	Inpatients from oncology, orthopedics, and urology wards	Denmark	81	Protein-enriched small dishes supplementary to standard food service, ONS or snacks	Standard hospital diet
Neelemaat et al,^24^ 2012	General medical inpatients (≥60 y of age)	The Netherlands	210	Energy- and protein-enriched diet, 2 additional servings of ONS, 2520 kJ/d (to convert to kcal, divide by 4.186), 24 g protein/d, orally 400 U Vitamin D_3_ and 500 mg calcium/d, telephone counseling	Usual care
Ollenschläger et al,^25^ 1992	Patients with induction treatment for leukemia	Germany	29	Menus of free choice, nutritional education, daily visits by the dietician, and record of food intake	Menus of free choice, no nutritional education
Potter et al,^26^ 2001 and Roberts et al,^27^ 2003	General geriatric inpatients	United Kingdom	381	120 mL oral sip-feed supplement 3/d, 540 kcal/d, 22.5 g protein	Normal hospital food
Rüfenacht et al,^28^ 2010	General medical inpatients	Switzerland	36	Individual nutritional plan with food enrichment, energy- and/or protein-rich snacks, beverages and energy-dense ONS	2 U ONS providing 200 mL each with 300 kcal and 12 g protein
Ryan et al,^29^ 2004	General medical inpatients (≥65 y of age)	France	16	Oral supplement (1050 kJ [to convert to kcal, divide by 4.186], 250 mL)	Standard hospital breakfast
Saudny-Unterberger et al,^30^ 1997	Inpatients with COPD exacerbation (40-85 y of age)	Canada	33	ONS, 39 kcal/kg/d	Standard food, 29 kcal/kg/d
Schuetz et al,^38^ 2019	General medical inpatients	Switzerland	2028	A systematic nutritional assessment by a dietitian was done to define nutritional targets, followed by individualized early nutritional support based on a previously published consensus algorithm and current nutritional guidelines	Standard nutritional management
Somanchi et al,^39^ 2011	General medical inpatients	United States	400	Nutritional screening of all patients, clinical nutritional plan initiated by the nurse manager	Usual hospital screening and nutritional counseling on demand
Starke et al,^40^ 2011	General medical inpatients	Switzerland	132	Individual nutritional care (food supply, fortification of meals with maltodextrins, rapeseed oil, cream and/or protein, powder, in-between snacks, and ONS); protein intake 1.0 g/kg body weight	Standard nutritional care, including prescription of ONS upon discretion of physician
Vermeeren et al,^31^ 2004	Inpatients with COPD exacerbation	The Netherlands	56	Liquid oral supplement 3x 125 mL, 2.38 MJ/d (to convert to kcal, divide by 0.0041858), 20 energy % protein, 20 energy % fat, and 60 energy % carbohydrate, standardized dietetic consultation	Free choice of normal hospital food and placebo 3 × 125 mL, 0 MJ/d
Vlaming et al,^32^ 2001	General medical, surgical, or orthopedic inpatients	United Kingdom	549	Normal hospital food plus 400 mL oral sip-feed supplement, 600 kcal/d, 25.0 g protein/d, 80.8 g carbohydrates/d, 19.6 g fat/d, multivitamins	Normal hospital food plus 400 mL placebo, 100 kcal/d, 25 g carbohydrates/d plus multivitamins
Volkert et al,^41^ 1996	General geriatric inpatients	Germany	72	Normal hospital food and 400 mL/d (2100 kJ [to convert to kcal divide by 4.186]) liquid supplement, 200 mL/d (1050 kJ) for the following 6 mo at home	Normal hospital food, usual care without supplements

Abbreviations: COPD, chronic obstructive pulmonary disease; ONS, oral nutrition supplements.

Assessment of risk of bias, which was performed as recommended by the Cochrane Collaboration (risk of bias graph in eFigure 2 in the Supplement), revealed that of the 27 studies, 17 had a low risk of random sequence generation and randomization concealment bias, 15 had a low risk of attrition bias for objective outcomes, and 19 had a low risk of reporting bias. Approximately 70% of the studies had high risk of performance bias for objective outcomes because blinding of participants and personnel was not undertaken. There was a large proportion of unclear risk of bias related to studies not reporting subjective outcomes. Other biases were not detected in most trials. Overall, risk of bias was less pronounced in the present study compared with the initial report,^9^ with newer trials showing better methodological quality. Funnel plots revealed no evidence of publication bias.

### Primary Outcome

**Figure 1.**
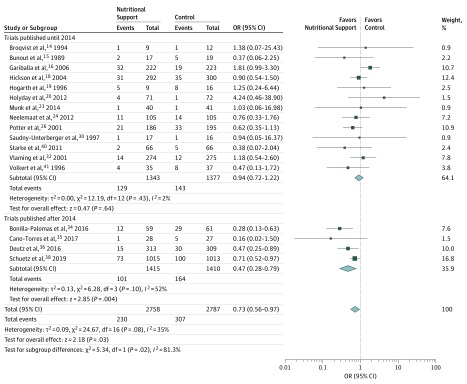
Forest Plot Comparing Nutritional Intervention vs Control for Mortality, Stratified by Publication Year A Mantel-Haenszel random-effects model was used. Squares indicate mean values, with the size of squares reflecting the weight and the lines indicating 95% CIs. Diamonds indicate pooled estimates, with horizontal points of the diamonds indicating 95% CIs. OR indicates odds ratio.

The analyses of the outcomes in the overall population and in subgroups are provided in Table 2. A total of 17 studies^14,15,16,18,19,20,23,24,26,30,32,34,35,36,38,40,41^ (13 older and 4 newer) reported data on mortality, the primary outcome. The association of the intervention with mortality risk for each trial, as well as the overall association stratified by newer vs older trials is shown in Figure 1. The mortality rate was 8.3% (230 of 2758) among the intervention group patients compared with 11.0% (307 of 2787) among the control group patients (OR, 0.73; 95% CI, 0.56-0.97, *P* = .03). There was a low level of heterogeneity among trials (*I*^2^ = 35%, *P* = .08) (Table 2). This significant reduction in mortality associated with the nutritional support was different from the nonsignificant association observed in the original meta-analysis (OR, 0.96; 95% CI, 0.72-1.27).^9^

**Table 2.** Outcome Analyses: Overall Population and Subgroups

Population/Variable	Mortality, OR (95% CI)	Infections, OR (95% CI)	Nonelective Readmissions, Risk Ratio (95% CI)	Mean Difference (95% CI)				
				Function, Barthel Index, Points	Length of Stay, d	Daily Energy Intake, kcal	Daily Protein Intake, g	Weight Change, kg
Overall population								
Intervention, events/total (%) or mean, No.	230/2758 (8.3)	88/1817 (4.8)	280/1903 (14.7)	17.3	11.5	1618	59	0.63
Control, events/total (%) or mean, No.	307/2787 (11.0)	102/1825 (5.6)	339/1880 (18.0)	16.9	12.0	1331	48	−0.19
Overall OR mean difference (95% CI)	0.73 (0.56 to 0.97)	0.86 (0.64 to 1.16)	0.76 (0.60 to 0.96)	0.32 (−0.51 to 1.15)	−0.24 (−0.58 to 0.09)	365 (272 to 458)	17.7 (12.1 to 23.3)	0.73 (0.32 to 1.13)
*I*^2^ Test for overall effect, %	35	0	48	77	0	84	88	100
Subgroup analysis stratified by degree of malnutrition								
Established malnutrition	0.52 (0.34 to 0.80)	NA	0.36 (0.20 to 0.64)	4.00 (1.69 to 6.31)	−2.08 (−4.19 to 0.02)	304 (218 to 389)	16.1 (5.1 to 27.1)	0.96 (0.42 to 1.50)
At nutritional risk	0.85 (0.62 to 1.18)	0.86 (0.64 to 1.15)	0.86 (0.74 to 1.00)	0.02 (−0.54 to 0.59)	−0.17 (−0.51 to 0.17)	394 (262 to 526)	16.3 (9.8 to 22.9)	0.86 (0.79 to 0.93)
*I*^2^ Test for subgroup difference, %	69	NA	88	91	68	21	0	0
Subgroup analysis stratified by mortality rate in control group								
High mortality (≥10%)	0.61 (0.43 to 0.87)	0.77 (0.17 to 3.46)	0.28 (0.12 to 0.65)	0.85 (−1.47 to 3.16)	−1.32 (−2.52 to −0.12)	231 (81 to 280)	16.0 (2.9 to 29.2)	0.14 (−0.61 to 0.88)
Low mortality (<10%)	0.91 (0.59 to 1.40)	0.86 (0.64 to 1.17)	0.86 (0.72 to 1.02)	0.14 (−0.70 to 0.98)	−0.12 (−0.49 to 0.24)	428 (316 to 540)	16.8 (9.9 to 23.6)	0.86 (0.79 to 0.93)
*I*^2^ Test for subgroup difference, %	49	0	85	0	71	77	0	73
Stratification by adherence to nutrition protocol								
High adherence	0.67 (0.54 to 0.84)	0.89 (0.62 to 1.26)	0.91 (0.76 to 1.10)	0.56 (0.07 to 1.05)	−0.17 (−0.52 to 0.19)	402 (313 to 491	19.6 (12.9 to 26.3)	0.87 (0.81 to 0.93)
Low adherence	0.88 (0.44 to 1.76)	0.79 (0.45 to 1.38)	0.58 (0.36 to 0.96)	0.33 (−0.88 to 1.55)	−0.82 (−1.80 to 0.16)	107 (24 to 191)	8.3 (−3.2 to 19.8)	−0.20 (−0.23 to −0.17)
*I*^2^ Test for subgroup difference, %	0	0	64	0	34	96	64	100
Stratification by route of nutritional support								
Oral routes	0.74 (0.58 to 0.93)	0.75 (0.50 to 1.11)	0.74 (0.56 to 0.99)	0.33 (−0.88 to 1.55)	−0.26 (−0.67 to 0.15)	367 (247 to 487)	16.2 (9.5 to 22.8)	0.761 (0.27 to 1.14)
Mixed routes	0.71 (0.52 to 0.97)	1.02 (0.65 to 1.61)	0.73 (0.35 to 1.53)	0.56 (0.07 to 1.05)	−0.98 (−3.32 to 1.36)	417 (108 to 727)	28.8 (−9.0 to 66.6)	0.90 (0.89 to 0.91)
*I*^2^ Test for subgroup difference, %	0	6	0	0	0	0	0	0
Stratification by publication year								
Older (2014 or earlier)	0.94 (0.72 to 1.22)	0.75 (0.50 to 1.11)	0.71 (0.57 to 0.87)	0.33 (−0.88 to 1.55)	−0.42 (−1.09 to 0.24)	396 (272 to 520)	18.5 (11.2 to 25.9)	0.66 (0.17 to 1.15)
Newer (2015 or later)	0.47 (0.28 to 0.79)	1.02 (0.65 to 1.61)	0.78 (0.50 to 1.22)	0.56 (0.07 to 1.05)	−0.27 (−0.87 to 0.33)	286 (239 to 333)	10.0 (8.1 to 11.9)	0.86 (0.78 to 0.95)
*I*^2^ Test for subgroup difference, %	81	6	0	0	0	62	79	0

Abbreviations: NA, not applicable; OR, odds ratio.

### Secondary Outcomes

**Figure 2.**
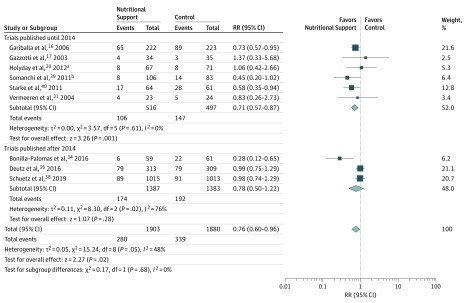
Forest Plot Comparing Nutritional Intervention vs Control for Nonelective Hospital Readmissions, Stratified by Publication Year A Mantel-Haenszel random-effects model was used. Squares indicate mean values, with the size of squares reflecting the weight and the lines indicating 95% CIs. Diamonds indicate pooled estimates, with horizontal points of the diamonds indicating 95% CIs. RR indicates risk ratio. ^a^Calculated and approximated from readmission frequency. ^b^Calculated and approximated from readmission rate.

Rates of nonelective hospital readmissions were reported in 9 studies^16,17,20,31,34,36,38,39,40^ (Table 2 and Figure 2). Compared with the control group, nutritional support interventions were associated with a significant reduction of nonelective hospital readmissions (14.7% [280 of 1903] in the intervention vs 18.0% [339 of 1880] in the control group; RR, 0.76; 95% CI, 0.60-0.96; *P* = .02), although there was heterogeneity among trials (*I*^2^ = 48%, *P* = .05). There was no statistically significant difference between the older and newer studies. The original meta-analysis^9^ had also reported an association between nutritional support and reduced nonelective hospital readmissions (RR, 0.71; 95% CI, 0.57-0.87).

Compared with the control group, the intervention group patients had no differences in rates for infections (4.8% [88 of 1817] vs 5.6% [102 of 1825]; OR, 0.86; 95% CI, 0.64-1.16), functional outcome at follow-up (17.3 vs 16.9 points; mean difference in Barthel index score, 0.32 points; 95% CI, −0.51 to 1.15), or LOS (11.5 days vs 12.0 days; mean difference, −0.24 days; 95% CI, −0.58 to 0.09) (Table 2 and eFigures 3, 4, and 5 in the Supplement).

Regarding nutritional outcomes (Table 2 and eFigures 6, 7, and 8 in the Supplement), nutritional support interventions were associated with a significantly higher energy intake (1618 kcal in the intervention group vs 1331 kcal in the control group; mean difference, 365 kcal; 95% CI, 272-458 kcal) and protein intake (59 g in the intervention group vs 48 g in the control group; mean difference, 17.7 g; 95% CI, 12.1-23.3 g). In addition, there was a significant increase in body weight (0.63 kg in the intervention group vs −0.19 kg in the control group; mean difference, 0.73 kg; 95% CI, 0.32-1.13 kg). Heterogeneity among trials was high (*I*^2^ = 84% [energy intake], *I*^2^ = 88% [protein intake], and *I*^2^ = 100% [weight change]).

### Sensitivity Analyses

Trials were stratified according to the degree of malnutrition, baseline mortality rate in the control group, adherence to the nutrition protocol, route of nutritional support, and publication year (before or after 2015) (Table 2).

The sensitivity analysis suggested a more pronounced reduction in the risk of mortality in recent trials (2015 or later) (OR, 0.47; 95% CI, 0.28-0.79) compared with that in older studies (OR, 0.94; 95% CI, 0.72-1.22), in patients with established malnutrition (OR, 0.52; 95% CI, 0.34-0.80) compared with that in patients at nutritional risk (OR, 0.85; 95% CI, 0.62-1.18), and in trials with high protocol adherence (OR, 0.67; 95% CI, 0.54-0.84) compared with that in trials with low protocol adherence (OR, 0.88; 95% CI, 0.44-1.76).

The results suggest larger benefits associated with nutritional support for the subgroup of patients with established malnutrition compared with that for the subgroup of patients at nutritional risk, particularly for functional outcome and nonelective hospital readmissions (and a beneficial association between nutritional support and mortality and LOS). Among the individuals with a higher mortality rate (≥10%) vs those with a lower mortality rate (<10%), the associations of the intervention were stronger. However, this effect was only significant for nonelective readmissions and energy intake.

There was no evidence of other associations in subgroup analyses based on protocol adherence or route of nutritional support except for energy intake and weight change, which was increased in the studies with high adherence to the nutrition protocol (energy intake [402 kcal]; weight change [0.87 kg]) compared with the studies with lower adherence (energy intake [107 kcal]; weight change [−0.20 kg]). Associations between nutritional support and mortality reduction and weight gain were more pronounced in newer studies compared with the older trials.

An additional sensitivity analysis was performed to better understand whether associations of nutritional support would be similar if the largest trial (EFFORT [Effect of Early Nutritional Support on Frailty, Functional Outcomes, and Recovery of Malnourished Medical Inpatients Trial]^38^) was excluded (Table 3). When excluding EFFORT trial data from the analysis, associations of nutritional support with mortality (OR, 0.73; 95% CI, 0.52-1.03), as well as nonelective hospital readmissions (RR, 0.71; 95% CI, 0.54-0.94), were similar.

**Table 3.** Outcome Analyses With and Without EFFORT^38^

Population/Variable	Mortality, OR (95% CI)	Infections, OR (95% CI)	Nonelective Readmissions, Risk Ratio (95% CI)	Mean Difference (95% CI)				
				Function, Barthel Index, Points	Length of Stay, d	Daily Energy Intake, kcal	Daily Protein Intake, g	Weight Change, kg
Overall population								
Intervention, events/total (%) or mean, No.	230/2758 (8.3)	88/1817 (4.8)	280/1903 (14.7)	17.3	11.5	1618	59	0.63
Control, events/total (%) or mean, No.	307/2787 (11.0)	102/1825 (5.6)	339/1880 (18.0)	16.9	12.0	1331	48	−0.19
Overall estimate	0.73 (0.56 to 0.97)	0.86 (0.64 to 1.16)	0.76 (0.60 to 0.96)	0.32 (−0.51 to 1.15)	−0.24 (−0.58 to 0.09)	365 (272 to 458)	17.7 (12.1 to 23.3)	0.73 (0.32 to 1.13)
*I*^2^ Test for overall effect, %	35	0	48	77	0	84	88	100
Overall population without EFFORT								
Intervention, events/total (%) or mean, No.	157/1743 (9.0)	48/802 (5.9)	191/888 (21.5)	15.5	12.8	1950	73	0.37
Control, events/total (%) or mean, No.	207/1774 (11.7)	63/812 (7.8)	248/867 (28.6)	14.8	14.0	1543	54	−0.21
Overall estimate	0.73 (0.52 to 1.03)	0.75 (0.50 to 1.11)	0.71 (0.54 to 0.94)	0.33 (−0.88 to 1.55)	−0.38 (−0.85 to 0.10)	382 (266 to 498)	18.5 (11.2 to 26.9)	0.71 (0.27 to 1.14)
*I*^2^ Test for overall effect, %	39	0	47	78	5	84	89	99

Abbreviations: EFFORT, Effect of Early Nutritional Support on Frailty, Functional Outcomes, and Recovery of Malnourished Medical Inpatients Trial; OR, odds ratio.

## Discussion

The findings of this updated systematic review and meta-analysis of RCTs investigating the association of nutritional support interventions with outcomes in medical inpatients who are malnourished or at nutritional risk were 3-fold. First, compared with the original meta-analysis^9^ that included trials published before April 2014 (9 trials), the 5 new trials were a higher quality, had lower bias, and collectively nearly doubled the total patient population studied in this updated meta-analysis (3736 patients from the original study plus 3067 patients from the 5 new studies). Newer trials also differed with regard to the nutritional interventions used, with a higher quality of protein^13^ and a more individualized, patient-specific approach. Second, our analysis suggests that nutritional support compared with no support was statistically significantly associated with increased protein and energy intake during the hospital stay, with an increased body weight. Third, our analysis found that nutritional support was associated with a statistically significant reduction in mortality and nonelective hospital readmissions and thus had favorable associations with clinical outcomes beyond the known associations with metabolic parameters.

There are important differences in the results between the original meta-analysis^9^ and the present updated analysis, particularly with regard to mortality. In the original analysis, the mortality difference was 0.5% in favor of nutritional support,^9^ whereas the absolute mortality benefit increased to 2.8% in the present updated analysis, corresponding to a number needed to treat of 36 to prevent 1 death. The inclusion of 2 recent, large, and high-quality RCTs—namely EFFORT^38^ and NOURISH (Nutrition Effect on Unplanned Readmissions and Survival in Hospitalized Patients)^36^ that reported lower mortality associated with nutritional support—may have contributed to this shift in results, although overall heterogeneity regarding the mortality outcome was only low to moderate. This finding suggests that the decreased risk of mortality may have been masked in older studies owing to small sample sizes (eg, 22 patients^14^), lower study quality, and quality of nutritional support used in trials.^9^ Overall, the decreased risk of mortality associated with nutritional support found in the present analysis suggests that malnutrition is a modifiable risk factor for mortality, with nutritional support being an effective treatment option.

These findings differ from those of other recent reviews of nutritional support. A recent Cochrane review^10^ did not find a positive association between nutritional support and outcomes in hospitalized adults at nutritional risk. However, this study included a larger variety of patients, including intensive care unit and surgical patients, who may have specific nutritional and metabolic needs. It should be noted that patients treated in intensive care units tend to be highly catabolic, and it is likely that nutritional support would not alter this process. On the other hand, nutritional support in non–critically ill medical patients who are malnourished may result in increased protein synthesis and increased lean body mass. The Cochrane review^10^ also included a wider range of interventions, including parenteral nutrition, which may be associated with a higher risk for adverse outcomes. Furthermore, the literature searches were conducted in February 2016, which excludes 2 recent, large, nutritional support RCTs of medical inpatients at nutritional risk (the EFFORT trial,^38^ published in 2018 with 2028 patients; and the NOURISH trial,^36^ published in 2016 with 652 patients). Inclusion of these trials may also alter the overall interpretation of this present study.

One could postulate that nutritional support would have limited the loss of lean body mass, thereby improving muscle strength and functional outcomes, but this finding was not observed in the present study. However, only 5 studies^16,18,19,38,41^ assessed functional outcomes, defined by the Barthel index score at follow-up (eFigure 4 in the Supplement). The absence of any association between nutritional support and improved functional outcomes may be attributable to the methods used in the few studies that assessed this outcome and the relatively short duration of nutritional support (or time for the assessment of functional status).

Of importance, in the present analysis, nutritional support was associated with more benefits in the subgroup of patients with established malnutrition vs than in the patients at nutritional risk, particularly for hospital readmissions, functional outcomes, LOS, and mortality, for which the differences between groups were statistically significant or more pronounced. This finding highlights the importance of using validated methods to assess patients’ nutritional status to identify those who are more likely to benefit from nutritional support. A team approach including nurses, dieticians, and physicians may provide a solution to the problem of identifying and appropriately addressing malnutrition in the hospital setting.

In the context of increasing health care costs, the significant reduction in hospital readmissions observed on the overall analysis and the reduction in LOS shown in the subgroup of patients with established malnutrition may be particularly relevant for policy makers. If these findings are borne out in subsequent trials, given that approximately 30% of general medical inpatients meet the criteria for malnutrition,^2^ patient-specific nutritional interventions may result in substantial cost and hospital utilization reductions in addition to the mortality benefits (eg, in an analysis of inpatient use of oral nutritional supplements in more than 1 million participants^42^). Future studies should focus on the cost-effectiveness of providing nutritional support interventions for medically ill patients. The evaluation of other patient-centered outcomes, such as quality of life, should also be explored in more detail.

### Limitations

This study has limitations. Several of the included studies had a high or unknown risk of bias, small sample sizes, and short study duration (ie, limited to the hospital stay). Malnutrition starts in the community (the patient is identified as being malnourished on admission to the hospital) and does not end at the hospital discharge; therefore, the causes of malnutrition in the community need to be explored, and nutritional support should be continued after hospital discharge. In addition, heterogeneity was observed with regard to the types of interventions and the control groups. Some trials were placebo-controlled efficacy trials focusing on the effect of specific products, whereas others were effectiveness trials comparing complex interventions with routine care, which may vary across health care settings.

## Conclusions

This updated systematic review and meta-analysis found that use of nutritional support interventions was associated with clinically significant improvements of important clinical outcomes in the medical inpatient population, in whom malnutrition is highly prevalent.^43^ This analysis supports the current practice guidelines issued by the European Society for Clinical Nutrition and Metabolism (ESPEN)^4^ and the American Society for Parenteral and Enteral Nutrition (ASPEN),^8^ advocating a proactive, screening-based approach for initiating nutritional support during the hospital stay of medical inpatients who are malnourished or at nutritional risk.

## Notes

Supplement. **eAppendix.** Search Strategy Used in MEDLINE **eFigure 1.** Flow Chart of Studies’ Selection **eFigure 2** Risk of Bias Overall and Stratified by Trial **eFigure 3.** Forest Plot Comparing Nutritional Intervention vs. Control for Infection Stratified by Publication Year **eFigure 4.** Forest Plot Comparing Nutritional Intervention vs. Control for Functional Outcome Stratified by Publication Year **eFigure 5.** Forest Plot Comparing Nutritional Intervention vs. Control for Length of Stay Stratified by Publication Year **eFigure 6.** Forest Plot Comparing Nutritional Intervention vs. Control for Daily Energy Intake Stratified by Publication Year **eFigure 7.** Forest Plot Comparing Nutritional Intervention vs. Control for Daily Protein Intake Stratified by Publication Year **eFigure 8.** Forest Plot Comparing Nutritional Intervention vs. Control for Weight Change Stratified by Publication Year **eTable.** Adherence to Study Protocol **eReferences.**
