# Supplementary material for: Association of Nutritional Support With Clinical Outcomes Among Medical Inpatients Who Are Malnourished or at Nutritional Risk: An Updated Systematic Review and Meta-analysis
Source: JAMA Netw Open. 2019 Nov 20;2(11):e1915138. doi: 10.1001/jamanetworkopen.2019.15138 (PMC6902795; doi:10.1001/jamanetworkopen.2019.15138)
Supplement: Supplement. — eAppendix. Search Strategy Used in MEDLINE eFigure 1. Flow Chart of Studies’ Selection eFigure 2 Risk of Bias Overall and Stratified by Trial eFigure 3. Forest Plot Comparing Nutritional Intervention vs. Control for Infection Stratified by Publication Year eFigure 4. Forest Plot Comparing Nutritional Intervention vs. Control for Functional Outcome Stratified by Publication Year eFigure 5. Forest Plot Comparing Nutritional Intervention vs. Control for Length of Stay Stratified by Publication Year eFigure 6. Forest Plot Comparing Nutritional Intervention vs. Control for Daily Energy Intake Stratified by Publication Year eFigure 7. Forest Plot Comparing Nutritional Intervention vs. Control for Daily Protein Intake Stratified by Publication Year eFigure 8. Forest Plot Comparing Nutritional Intervention vs. Control for Weight Change Stratified by Publication Year eTable. Adherence to Study Protocol eReferences. [file jamanetwopen-2-e1915138-s001.pdf]

Supplementary Online Content

Gomes F, Baumgartner A, Bounoure L, et al. Association of nutritional support with clinical outcomes among medical inpatients who are malnourished or at nutritional risk: an updated systematic review and meta-analysis. *JAMA Netw Open.* 2019;2(11): e1915138. doi:10.1001/jamanetworkopen.2019.15138

eAppendix. Search Strategy Used in MEDLINE
eFigure 1. Flow Chart of Studies' Selection
eFigure 2. Risk of Bias Overall and Stratified by Trial
eFigure 3. Forest Plot Comparing Nutritional Intervention vs. Control for Infection Stratified by Publication Year
eFigure 4. Forest Plot Comparing Nutritional Intervention vs. Control for Functional Outcome Stratified by Publication Year
eFigure 5. Forest Plot Comparing Nutritional Intervention vs. Control for Length of Stay Stratified by Publication Year
eFigure 6. Forest Plot Comparing Nutritional Intervention vs. Control for Daily Energy Intake Stratified by Publication Year
eFigure 7. Forest Plot Comparing Nutritional Intervention vs. Control for Daily Protein Intake Stratified by Publication Year
eFigure 8. Forest Plot Comparing Nutritional Intervention vs. Control for Weight Change Stratified by Publication Year
eTable. Adherence to Study Protocol
eReferences.

This supplementary material has been provided by the authors to give readers additional information about their work.

eAppendix. Search Strategy Used in MEDLINE

1. exp *PROTEIN-ENERGY MALNUTRITION/ or malnutrition.mp. or exp *MALNUTRITION/
2. maln*.mp. [mp=title, abstract, original title, name of substance word, subject heading word, keyword heading word, protocol supplementary concept word, rare disease supplementary concept word, unique identifier, synonyms]
3. undern*.mp. [mp=title, abstract, original title, name of substance word, subject heading word, keyword heading word, protocol supplementary concept word, rare disease supplementary concept word, unique identifier, synonyms]
4. 1 or 2 or 3
5. exp *Nutrition Therapy/ or exp *Enteral Nutrition/ or nutritional therapy.mp.
6. exp *Nutritional Support/ or nutrition support.mp.
7. (nutrition* adj3 (support or therapy)).mp. [mp=title, abstract, original title, name of substance word, subject heading word, keyword heading word, protocol supplementary concept word, rare disease supplementary concept word, unique identifier, synonyms]
8. dietary advice.mp. [mp=title, abstract, original title, name of substance word, subject heading word, keyword heading word, protocol supplementary concept word, rare disease supplementary concept word, unique identifier, synonyms]
9. food fortification.mp. [mp=title, abstract, original title, name of substance word, subject heading word, keyword heading word, protocol supplementary concept word, rare disease supplementary concept word, unique identifier, synonyms]
10. oral nutrition* supplement*.mp. [mp=title, abstract, original title, name of substance word, subject heading word, keyword heading word, protocol supplementary concept word, rare disease supplementary concept word, unique identifier, synonyms]
11. (enteral adj1 (nutrition or feeding)).mp. [mp=title, abstract, original title, name of substance word, subject heading word, keyword heading word, protocol supplementary concept word, rare disease supplementary concept word, unique identifier, synonyms]
12. 5 or 6 or 7 or 8 or 9 or 10 or 11
13. hospital.mp. or exp *Hospitals/
14. hospital*.mp. [mp=title, abstract, original title, name of substance word, subject heading word, keyword heading word, protocol supplementary concept word, rare disease supplementary concept word, unique identifier, synonyms]
15. ward*.mp. [mp=title, abstract, original title, name of substance word, subject heading word, keyword heading word, protocol supplementary concept word, rare disease supplementary concept word, unique identifier, synonyms]
16. in?patient*.mp. [mp=title, abstract, original title, name of substance word, subject heading word, keyword heading word, protocol supplementary concept word, rare disease supplementary concept word, unique identifier, synonyms]
17. 13 or 14 or 15 or 16
18. 4 and 12 and 17
19. limit 18 to yr="2015 - 2019"

© 2019 Gomes F et al. *JAMA Network Open.*

eFigure 1. Flow Chart of Studies' Selection

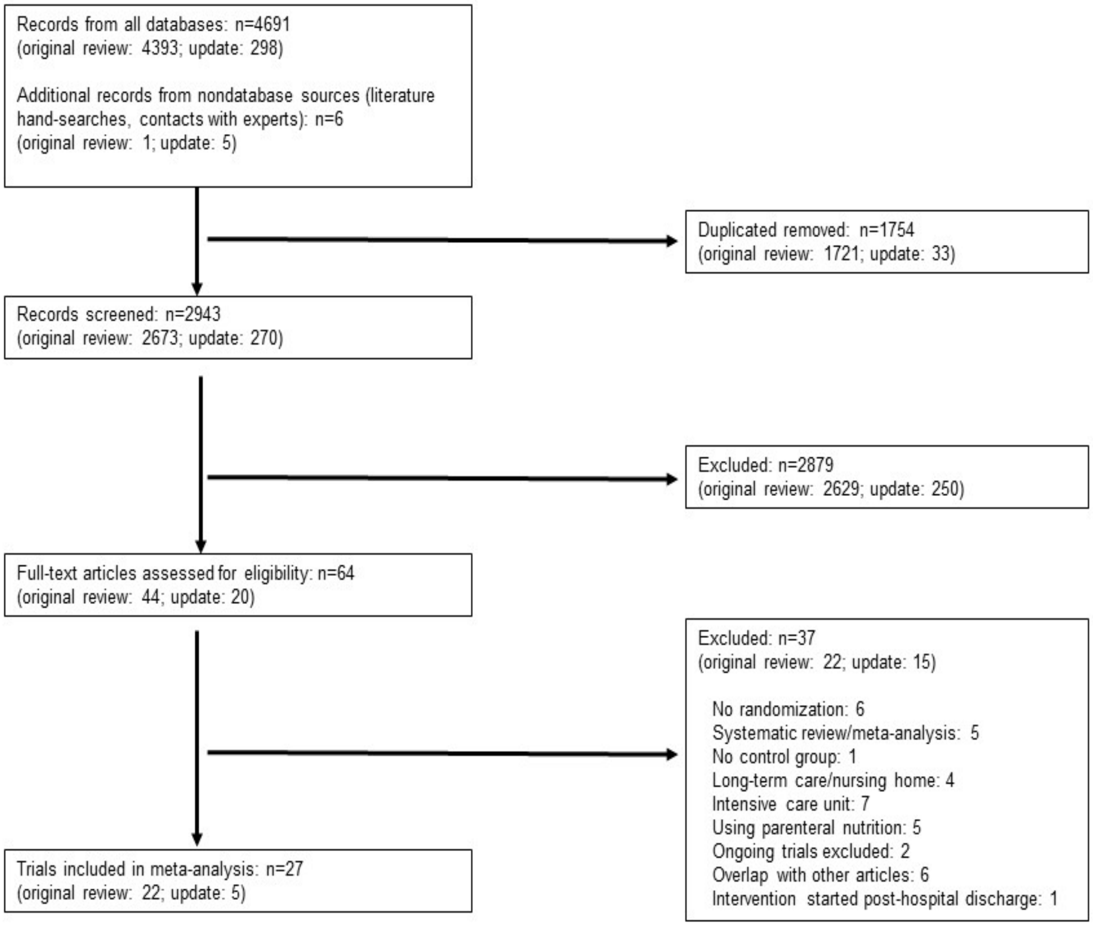

© 2019 Gomes F et al. *JAMA Network Open.*

eFigure 2. Risk of Bias Overall and Stratified by Trial

eFigure 2A. Risk of Bias Graph Overall

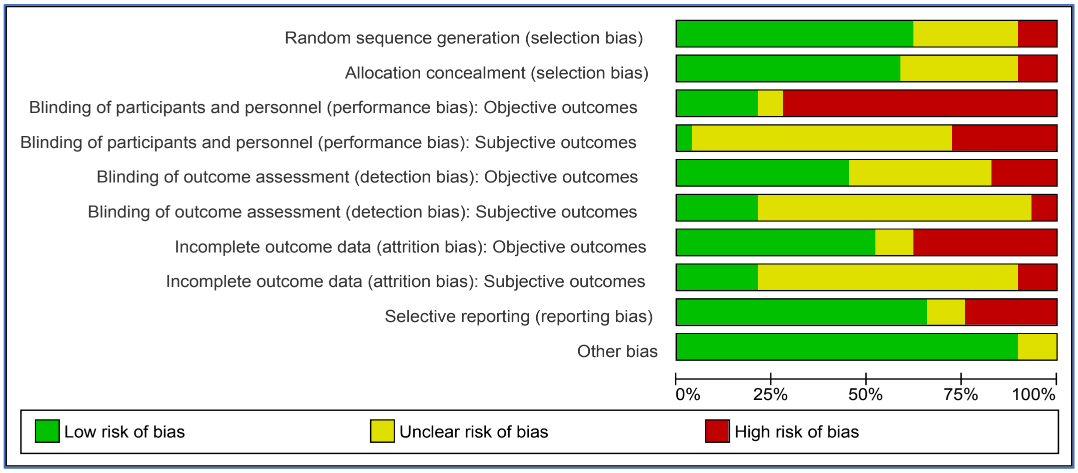

Review authors' judgements about each risk of bias item presented as percentages across all included studies

© 2019 Gomes F et al. *JAMA Network Open.*

eFigure 2B. Risk of Bias Graph Stratified by Trial

	Random sequence generation (selection bias)	Allocation concealment (selection bias)	Blinding of participants and personnel (performance bias): Objective outcomes	Blinding of participants and personnel (performance bias): Subjective outcomes	Blinding of outcome assessment (detection bias): Objective outcomes	Blinding of outcome assessment (detection bias): Subjective outcomes	Incomplete outcome data (attrition bias): Objective outcomes	Incomplete outcome data (attrition bias): Subjective outcomes	Selective reporting (reporting bias)	Other bias
Bonilla-Palomas 2016	?	?	−	?	?	?	+	?	+	+
Broqvist 1994	?	?	+	?	+	?	?	?	+	+
Bunout 1989	?	?	−	?	?	?	+	?	−	?
Cano-Torres 2017	+	?	−	?	?	?	+	?	+	+
Deutz 2016	+	+	+	+	+	+	+	+	?	+
Feldblum 2011	−	−	−	?	+	?	−	?	+	+
Gariballa 2006	+	+	+	?	+	?	−	?	+	+
Gazzotti 2003	+	+	−	?	−	?	−	?	−	+
Hickson 2004	+	+	−	?	−	?	+	?	?	+
Hogarth 1996	?	?	?	?	?	?	+	+	+	+
Holyday 2011	+	+	−	−	+	?	−	?	−	+
Huynh 2015	+	+	−	−	−	−	+	+	+	+
McEvoy 1982	?	?	−	?	?	?	+	?	+	+
McWhirter 1996	?	?	−	?	?	?	+	?	?	+
Munk 2014	+	+	−	?	+	?	−	?	−	+
Neelemaat 2012	+	+	−	−	+	+	?	?	+	+
Neelemaat 2012b	+	+	+	?	+	?	−	?	−	+
Ollenschlager 1992	?	?	−	?	−	−	?	?	+	+
Potter 2001	+	+	−	−	+	+	+	+	+	+
Roberts 2003	+	+	−	?	+	?	+	?	+	+
Rufenacht 2010	+	+	−	−	?	?	−	−	+	+
Ryan 2004	+	+	?	?	+	+	−	−	+	?
Saudny-Unterberger 1997	?	?	−	−	?	?	−	−	−	+
Schuetz 2019	+	+	−	−	+	+	+	+	+	+
Somanchi 2011	−	−	−	?	?	?	−	?	−	?
Starke 2011	+	+	−	−	−	+	+	+	+	+
Vermeeren 2004	−	−	+	?	?	?	+	?	+	+
Vlaming 2001	+	+	+	?	+	?	+	?	+	+
Volkert 1996	+	+	−	?	?	?	−	?	+	+

© 2019 Gomes F et al. *JAMA Network Open.*

eFigure 3. Forest Plot Comparing Nutritional Intervention vs. Control for Infection Stratified by Publication Year

	Nutritional support		Control			Odds Ratio
**Study or Subgroup**	**Events**	**Total**	**Events**	**Total**	**Weight**	**M-H, Random, 95% CI**
**6.2.1 ≤ 2014**						
Gariballa 2006	21	222	26	223	23.9%	0.79 [0.43, 1.45]
Gazzotti 2003	21	222	26	223	23.9%	0.79 [0.43, 1.45]
Hickson 2004 (1)	3	292	4	300	3.9%	0.77 [0.17, 3.46]
Starke 2011 (2)	3	66	7	66	4.5%	0.40 [0.10, 1.62]
**Subtotal (95% CI)**		**802**		**812**	**56.3%**	**0.75 [0.50, 1.11]**
Total events	48		63			
Heterogeneity: Tau² = 0.00; Chi² = 0.83, df = 3 (P = 0.84); I² = 0%						
Test for overall effect: Z = 1.44 (P = 0.15)						
**6.2.2 ≥ 2015**						
Schuetz 2019	40	1015	39	1013	43.7%	1.02 [0.65, 1.61]
**Subtotal (95% CI)**		**1015**		**1013**	**43.7%**	**1.02 [0.65, 1.61]**
Total events	40		39			
Heterogeneity: Not applicable						
Test for overall effect: Z = 0.11 (P = 0.92)						
**Total (95% CI)**		**1817**		**1825**	**100.0%**	**0.86 [0.64, 1.16]**
Total events	88		102			
Heterogeneity: Tau² = 0.00; Chi² = 1.89, df = 4 (P = 0.76); I² = 0%						
Test for overall effect: Z = 1.01 (P = 0.31)						
Test for subgroup differences: Chi² = 1.06, df = 1 (P = 0.30), I² = 5.5%						

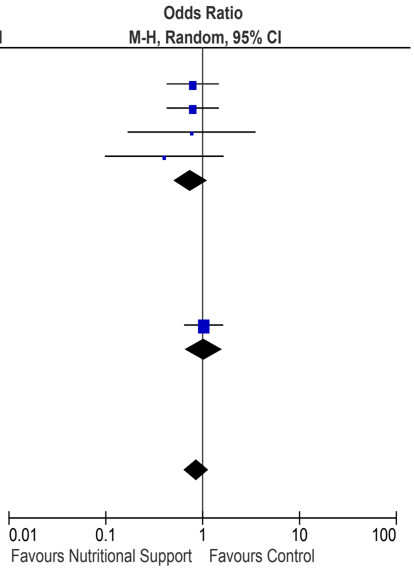

Footnotes
(1) use of antibiotics
(2) infections, NOT us of antibiotics (number als lower in intervention and higher in control group)

© 2019 Gomes F et al. *JAMA Network Open.*

eFigure 4. Forest Plot Comparing Nutritional Intervention vs. Control for Functional Outcome Stratified by Publication Year

	Nutritional support			Control				Mean Difference
**Study or Subgroup**	**Mean**	**SD**	**Total**	**Mean**	**SD**	**Total**	**Weight**	**IV, Random, 95% CI**
**6.4.1 ≤ 2014**								
Gariballa 2006	18.3	3	222	18.6	3	223	27.3%	-0.30 [-0.86, 0.26]
Hickson 2004 (1)	16	4.44	212	16	4.8	217	23.1%	0.00 [-0.87, 0.87]
Hogarth 1996	13.67	4.87	46	14.64	3.85	41	12.4%	-0.97 [-2.81, 0.87]
Volkert 1996 (2)	14	3.5	20	10	4.5	26	9.1%	4.00 [1.69, 6.31]
**Subtotal (95% CI)**			**500**			**507**	**71.9%**	**0.33 [-0.88, 1.55]**
Heterogeneity: Tau² = 1.06; Chi² = 13.49, df = 3 (P = 0.004); I² = 78%								
Test for overall effect: Z = 0.54 (P = 0.59)								
**6.4.2 ≥ 2015**								
Schuetz 2019	17.6	5.2	1015	17.04	5.96	1013	28.1%	0.56 [0.07, 1.05]
**Subtotal (95% CI)**			**1015**			**1013**	**28.1%**	**0.56 [0.07, 1.05]**
Heterogeneity: Not applicable								
Test for overall effect: Z = 2.25 (P = 0.02)								
**Total (95% CI)**			**1515**			**1520**	**100.0%**	**0.32 [-0.51, 1.15]**
Heterogeneity: Tau² = 0.58; Chi² = 17.33, df = 4 (P = 0.002); I² = 77%								
Test for overall effect: Z = 0.76 (P = 0.45)								
Test for subgroup differences: Chi² = 0.11, df = 1 (P = 0.74), I² = 0%								

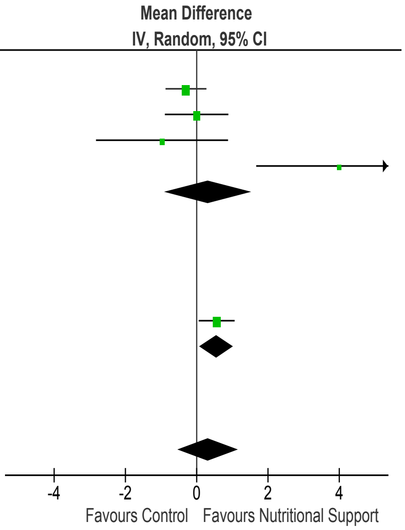

Footnotes
(1) SD approximated from interquartile range
(2) results combined for patients with good and bad compliance; mean and SD approximated; Barthel 100 scale adapted to Barthel 20 points scale

© 2019 Gomes F et al. *JAMA Network Open.*

eFigure 5. Forest Plot Comparing Nutritional Intervention vs. Control for Length of Stay Stratified by Publication Year

	Nutritional support			Control				Mean Difference
Study or Subgroup	Mean	SD	Total	Mean	SD	Total	Weight	IV, Random, 95% CI
**6.5.1 ≤ 2014**								
Bunout 1989 (1)	20	12.3	17	28	17.4	19	0.1%	-8.00 [-17.77, 1.77]
Gariballa 2006	9.4	7	222	10.1	8	223	5.8%	-0.70 [-2.10, 0.70]
Gazzotti 2003	19.8	15.1	35	21.1	10.1	35	0.3%	-1.30 [-7.32, 4.72]
Hickson 2004 (2)	21	17	292	23	18.5	300	1.4%	-2.00 [-4.86, 0.86]
Hogarth 1996	11.1	5.4	46	10.6	5.5	41	2.1%	0.50 [-1.80, 2.80]
Holyday 2011 (3)	13.7	11.7966	71	13.5	11.0309	72	0.8%	0.20 [-3.54, 3.94]
Munk 2014	10	8	41	10	8	40	0.9%	0.00 [-3.48, 3.48]
Neelemaat 2012	13	16.8	105	14	12.5	105	0.7%	-1.00 [-5.01, 3.01]
Potter 2001	16	34.5	165	18	18.5	162	0.3%	-2.00 [-7.99, 3.99]
Somanchi 2011	6.11	5.4	106	8.71	11.7	83	1.5%	-2.60 [-5.32, 0.12]
Starke 2011	17	10.4	66	18.6	17.1	66	0.5%	-1.60 [-6.43, 3.23]
Vermeeren 2004	9	2	29	9	2	27	10.2%	0.00 [-1.05, 1.05]
Vlaming 2001	14.2	24.9	275	11.4	16.4	174	0.8%	2.80 [-1.02, 6.62]
**Subtotal (95% CI)**			**1470**			**1347**	**25.4%**	**-0.42 [-1.09, 0.24]**
Heterogeneity: Tau² = 0.00; Chi² = 10.89, df = 12 (P = 0.54); I² = 0%								
Test for overall effect: Z = 1.25 (P = 0.21)								
**6.5.2 ≥ 2015**								
Cano-Torres 2017	6.4	3	28	8.4	4	27	3.2%	-2.00 [-3.87, -0.13]
Deutz 2016	5	3.2	313	5.1	3.6	309	37.9%	-0.10 [-0.64, 0.44]
Schuetz 2019	9.5	7	1015	9.6	6.1	1013	33.5%	-0.10 [-0.67, 0.47]
**Subtotal (95% CI)**			**1356**			**1349**	**74.6%**	**-0.27 [-0.87, 0.33]**
Heterogeneity: Tau² = 0.13; Chi² = 3.78, df = 2 (P = 0.15); I² = 47%								
Test for overall effect: Z = 0.89 (P = 0.38)								
**Total (95% CI)**			**2826**			**2696**	**100.0%**	**-0.24 [-0.58, 0.09]**
Heterogeneity: Tau² = 0.00; Chi² = 15.07, df = 15 (P = 0.45); I² = 0%								
Test for overall effect: Z = 1.42 (P = 0.16)								
Test for subgroup differences: Chi² = 0.11, df = 1 (P = 0.74), I² = 0%								

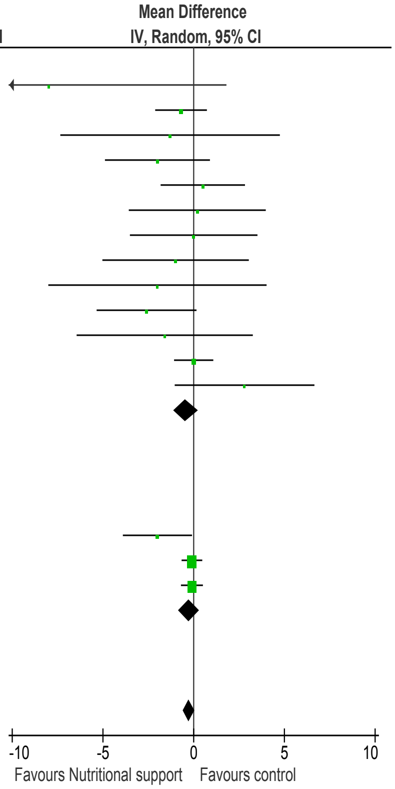

Footnotes
(1) SD approximated from SE
(2) SD approximated from IQR
(3) SD approximated from SE

© 2019 Gomes F et al. *JAMA Network Open.*

eFigure 6. Forest Plot Comparing Nutritional Intervention vs. Control for Daily Energy Intake Stratified by Publication Year

Study or Subgroup	Nutritional support Mean	SD	Total	Control Mean	SD	Total	Weight	Mean Difference IV, Random, 95% CI
**6.6.1 ≤ 2014**								
Broqvist 1994 (1)	2,785	727.6	7	2,205	609.7	12	1.7%	580.00 [-59.94, 1219.94]
Bunout 1989 (2)	2,707	292.7	17	1,813	527.4	19	5.4%	894.00 [619.05, 1168.95]
Gazzotti 2003 (3)	1,899	570	16	1,049	253	10	4.6%	850.00 [529.70, 1170.30]
Hickson 2004 (4)	1,381	498.032725	212	1,292	406.47745	217	9.4%	89.00 [2.86, 175.14]
McWhirter 1996 (5)	1,863	498.03	25	1,250	406.47745	13	5.0%	613.00 [318.15, 907.85]
Munk 2014	1,396	396	41	1,230	438	40	7.4%	166.00 [-15.98, 347.98]
Neelemaat 2012b	2,152	752	75	1,766	661	75	6.4%	386.00 [159.41, 612.59]
Potter 2001	1,409	482	46	1,090	417	48	7.4%	319.00 [136.47, 501.53]
Rufenacht 2010	1,845	439	18	1,425	446	18	5.1%	420.00 [130.90, 709.10]
Ryan 2004 (6)	1,657	393.66	8	1,452	373	8	3.8%	205.00 [-170.79, 580.79]
Saudny-Unterberger 1997 (7)	2,370	467.7072	14	2,015	332.0392	10	4.6%	355.00 [35.04, 674.96]
Starke 2011	1,553	116	66	1,115	145	66	10.0%	438.00 [393.20, 482.80]
Vermeeren 2004	2,601	480	23	2,245	607	24	4.8%	356.00 [43.82, 668.18]
Volkert 1996 (8)	1,201.5	316	20	995	409	26	6.7%	206.50 [-3.01, 416.01]
**Subtotal (95% CI)**			**588**			**586**	**82.5%**	**395.67 [271.49, 519.86]**
Heterogeneity: Tau² = 38427.46; Chi² = 83.69, df = 13 (P < 0.00001); I² = 84%								
Test for overall effect: Z = 6.24 (P < 0.00001)								
**6.6.2 ≥ 2015**								
Huynh 2015	2,436	650	104	2,200	637	103	7.5%	236.00 [60.67, 411.33]
Schuetz 2019	1,501	595.7	1015	1,211	517.3	1013	10.0%	290.00 [241.44, 338.56]
**Subtotal (95% CI)**			**1119**			**1116**	**17.5%**	**286.15 [239.36, 332.95]**
Heterogeneity: Tau² = 0.00; Chi² = 0.34, df = 1 (P = 0.56); I² = 0%								
Test for overall effect: Z = 11.99 (P < 0.00001)								
**Total (95% CI)**			**1707**			**1702**	**100.0%**	**364.80 [271.91, 457.69]**

Heterogeneity: Tau² = 21872.25; Chi² = 91.68, df = 15 (P < 0.00001); I² = 84%
Test for overall effect: Z = 7.70 (P < 0.00001)
Test for subgroup differences: Chi² = 2.62, df = 1 (P = 0.11), I² = 61.8%

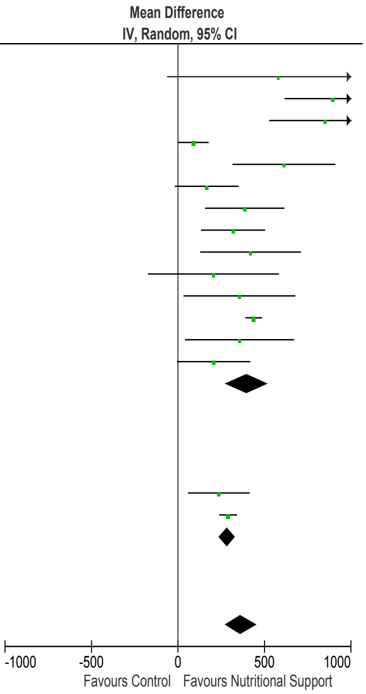

Footnotes
(1) SD approximated from SEM
(2) SD approximated from SEM
(3) intervention: spontaneous intake and intake from supplements combined
(4) SD approximated from median SD other included studies
(5) second intervention group compared to 50% of control group (see also oral feeding only vs no support), SD approximated from median SD other included studies
(6) 2 intervention groups combined, cross over design, 16 patients in total, analysed with 8/8
(7) SD approximated from SEM
(8) 2 intervention groups pooled

© 2019 Gomes F et al. *JAMA Network Open.*

eFigure 7. Forest Plot Comparing Nutritional Intervention vs. Control for Daily Protein Intake Stratified by Publication Year

	Nutritional support			Control				Mean Difference
Study or Subgroup	Mean	SD	Total	Mean	SD	Total	Weight	IV, Random, 95% CI
**6.7.1 ≤ 2014**								
Broqvist 1994 (1)	91	18.5203	7	74	20.7846	12	4.8%	17.00 [-1.07, 35.07]
Bunout 1989 (2)	80	12.3693	17	47	16.5638	19	7.5%	33.00 [23.51, 42.49]
Gazzotti 2003 (3)	69.4	21.2244381	16	37.2	9.9	10	6.6%	32.20 [20.12, 44.28]
Hickson 2004 (4)	50	19.893323	212	47	17.77821538	217	9.2%	3.00 [-0.57, 6.57]
McWhirter 1996 (5)	88.1	19.893323	25	39.5	17.77821538	13	6.5%	48.60 [36.18, 61.02]
Munk 2014	53	16	41	43	17	40	8.3%	10.00 [2.81, 17.19]
Neelemaat 2012b	78	34	75	63	30	75	7.3%	15.00 [4.74, 25.26]
Rufenacht 2010	62.2	18.2	18	50.7	16.5	18	6.9%	11.50 [0.15, 22.85]
Ryan 2004 (6)	58.5	17.5	8	62	19	8	4.8%	-3.50 [-21.40, 14.40]
Saudny-Unterberger 1997 (7)	94	22.4499	14	84	18.9737	10	5.2%	10.00 [-6.63, 26.63]
Starke 2011	65.4	16.4	66	43.9	17.2	66	8.7%	21.50 [15.77, 27.23]
Vermeeren 2004 (8)	105.25	23.39	23	78.94	18.22	24	6.6%	26.31 [14.29, 38.33]
Volkert 1996 (9)	47.575	13.55	20	32.8	14.1	26	8.0%	14.78 [6.74, 22.81]
**Subtotal (95% CI)**			**542**			**538**	**90.5%**	**18.54 [11.15, 25.94]**
Heterogeneity: Tau² = 151.44; Chi² = 106.12, df = 12 (P < 0.00001); I² = 89%								
Test for overall effect: Z = 4.91 (P < 0.00001)								
**6.7.2 ≥ 2015**								
Schuetz 2019	56.9	23	1015	46.9	20.9	1013	9.5%	10.00 [8.09, 11.91]
**Subtotal (95% CI)**			**1015**			**1013**	**9.5%**	**10.00 [8.09, 11.91]**
Heterogeneity: Not applicable								
Test for overall effect: Z = 10.25 (P < 0.00001)								
**Total (95% CI)**			**1557**			**1551**	**100.0%**	**17.69 [12.09, 23.28]**
Heterogeneity: Tau² = 84.95; Chi² = 111.25, df = 13 (P < 0.00001); I² = 88%								
Test for overall effect: Z = 6.20 (P < 0.00001)								
Test for subgroup differences: Chi² = 4.80, df = 1 (P = 0.03), I² = 79.2%								

Mean Difference
IV, Random, 95% CI

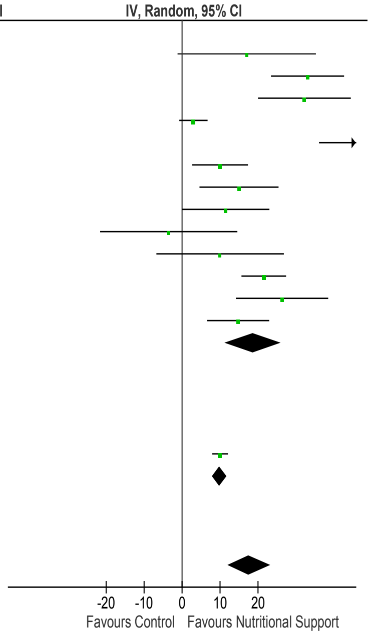

Footnotes

(1) SD approximated from SEM
(2) approximated from SEM
(3) SD intervention group combined spontaneous intake and supplement intake
(4) SD approximated from median SD other included studies
(5) second intervention group compared to 50% of control group, SD approximated from median SD other included studies
(6) 2 intervention groups pooled, cross over design, 16 patients in total, analysed with 8/8
(7) approximated from SEM
(8) protein intake approximated based on calculated body weight (from weight change)
(9) intervention: groups combined (good and bad supplement acceptance)

© 2019 Gomes F et al. *JAMA Network Open.*

eFigure 8. Forest Plot Comparing Nutritional Intervention vs. Control for Weight Change Stratified by Publication Year

Study or Subgroup	Nutritional support Mean	SD	Total	Control Mean	SD	Total	Weight	Mean Difference IV, Random, 95% CI
**6.8.1 ≤ 2014**								
Broqvist 1994 (1)	0.8	2.98727273	7	-0.2	2.55229091	12	1.9%	1.00 [-1.64, 3.64]
Bunout 1989 (2)	-6.29	6.1847	17	-4.65	7.846	19	0.7%	-1.64 [-6.23, 2.95]
Gariballa 2006 (3)	3.3	2.98727273	119	2.3	2.55229091	106	7.0%	1.00 [0.28, 1.72]
Gazzotti 2003	0.28	3.8	35	-1.23	2.5	34	4.0%	1.51 [-0.00, 3.02]
Hickson 2004 (4)	-0.3	0.15	212	-0.1	0.125	217	9.0%	-0.20 [-0.23, -0.17]
Hogarth 1996	0.1	3.2	46	-0.5	3.6	41	4.2%	0.60 [-0.84, 2.04]
Holyday 2011 (5)	-0.9	3.6497	37	-0.9	2.2627	32	4.3%	0.00 [-1.41, 1.41]
Munk 2014	0.4	2.6	41	-0.4	1.8	40	5.9%	0.80 [-0.17, 1.77]
Neelemaat 2012b (6)	3.7	2.98727273	75	2.1	2.55229091	75	6.3%	1.60 [0.71, 2.49]
Potter 2001	0.4	2.6	142	-0.5	2.9	151	7.4%	0.90 [0.27, 1.53]
Rufenacht 2010 (7)	1.3	2.98727273	18	0.4	2.55229091	18	3.2%	0.90 [-0.92, 2.72]
Saudny-Unterberger 1997 (8)	0.209	2.5443	14	-0.078	0.6325	10	4.4%	0.29 [-1.10, 1.68]
Starke 2011	0	2.9	66	-1.4	3.2	66	5.6%	1.40 [0.36, 2.44]
Vermeeren 2004	1.73	1.3	23	1.12	1.2	24	7.0%	0.61 [-0.11, 1.33]
Volkert 1996 (9)	-0.94	2.98727273	20	-0.1	2.55229091	26	3.6%	-0.84 [-2.48, 0.80]
**Subtotal (95% CI)**			**872**			**871**	**74.5%**	**0.66 [0.17, 1.15]**
Heterogeneity: Tau² = 0.54; Chi² = 65.41, df = 14 (P < 0.00001); I² = 79%								
Test for overall effect: Z = 2.64 (P = 0.008)								
**6.8.2 ≥ 2015**								
Deutz 2016	0.55	0.32	313	-0.26	0.32	309	9.0%	0.81 [0.76, 0.86]
Huynh 2015	2	2.38	76	0.9	1.34	82	7.5%	1.10 [0.49, 1.71]
Schuetz 2019	0.4	0.1	1015	-0.5	0.2	1013	9.0%	0.90 [0.89, 0.91]
**Subtotal (95% CI)**			**1404**			**1404**	**25.5%**	**0.86 [0.78, 0.95]**
Heterogeneity: Tau² = 0.00; Chi² = 11.88, df = 2 (P = 0.003); I² = 83%								
Test for overall effect: Z = 19.98 (P < 0.00001)								
**Total (95% CI)**			**2276**			**2275**	**100.0%**	**0.73 [0.32, 1.13]**
Heterogeneity: Tau² = 0.47; Chi² = 5367.05, df = 17 (P < 0.00001); I² = 100%								
Test for overall effect: Z = 3.52 (P = 0.0004)								
Test for subgroup differences: Chi² = 0.65, df = 1 (P = 0.42), I² = 0%								

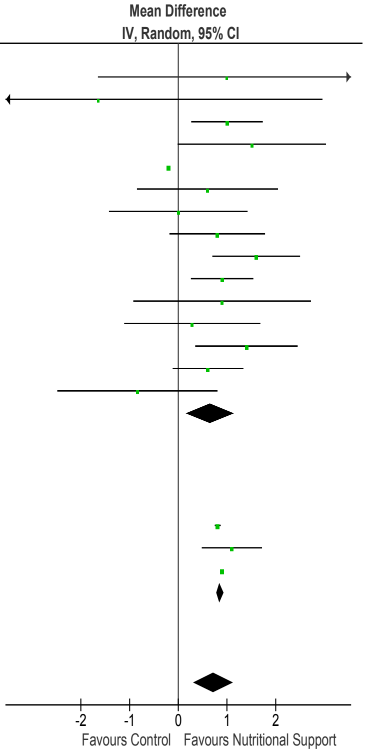

Footnotes
(1) SD approximated from median SD of other publications
(2) SD approximated from SEM
(3) calculated from baseline-weight and weight at follow up, SD approximated from SD other publications
(4) SD approximated from interquartile range
(5) SD approximated from SE
(6) mean calculated fromweight at baseline and follow up, SD approximated from SD other publications
(7) SD estimated from median SD of other publications
(8) SD approximated from SEM
(9) SD approximated from SD other publications

© 2019 Gomes F et al. *JAMA Network Open.*

eTable. Adherence to Study Protocol

First author, and year of publication	Compliance rate (in %)		Method for measurement of energy and protein intakes		Patients achieving energy and protein targets (in %)		Reasons behind a reduced compliance or efficiency		Adherence (final judgment)	Comments to author judgement
	INTERVENTION	*CONTROL*	*INTERVENTION*	*CONTROL*	*INTERVENTION*	*CONTROL*	*INTERVENTION*	*CONTROL*		
**Bonilla-Paloma 2016 (1)**	NR	NR	NR Nutritional intake was only assessed at baseline	NR Nutritional intake was only assessed at baseline	NR Nutritional intake was only assessed at baseline	NR Nutritional intake was only assessed at baseline	NR	NR	Poor	
**Broqvist 1994 (2)**	- Meals: NR<br>- ONS: 76.8% of total ONS offered consumed	- Meals: NR<br>- Placebo: 89.3% of total consumed	- Meals: dietary history (questionnaires)<br>- ONS: counting number of bottles consumed	- Meals: dietary history (questionnaires)<br>- ONS: counting number of bottles consumed	50% of patients achieving energy target (protein: NR)	14% of patients achieving energy target (protein: NR)	NR	NR	Good	
**Bunout 1989 (3)**	- Meals: NR<br>- Casein-based product: NR	- Meals: NR	- Meals: Weighing of food trays<br>- Casein-based product: NR	- Meals: Weighing of food trays	NR	NR	NR	NR	Good	Energy intake in intervention group approximately1000 kcal/d more than in control group
**Cano-Torres 2017 (4)**	- adherence to diet during hospital stay: 90.1%	- adherence to diet during hospital stay: 77.7%	Adherence to dietary intake was assessed by 24-hour recording.	Adherence to dietary intake was assessed by 24-hour recording	NR Total percentage of energy intake was not available	NR Total percentage of energy intake was not available	NR	NR	Good	The dietary advice received included motivation to adhere to a diet.
**Deutz 2016 (5)**	- Meals: NR<br>- ONS: mean expected intake was 73.2% in-hospital and 76.9% at 30-days post-discharge	- Meals: NR<br>- ONS: mean expected intake was 72.7% in-hospital and 78.4% at 30-days post-dsicharge	NR	NR	NR	NR	Reasons may be dependent on appetite, other clinical variables, or on reimbursement policies	Reasons may be dependent on appetite, other clinical variables, or on reimbursement policies	Good	The greater increases in body weight and serum 25-hydroxyvitamin D concentration among the intervention patients can be considered indicative of adherence with product intake
**Feldblum 2011 (6)**	NR	NR	24- hour recall (data not shown for assessment by Food Frequency Questionnaire)	24- hour recall (data not shown for assessment by Food Frequency Questionnaire)	NR	NR	NR	NR	Poor	No significant differences in intake between intervention and control group
**Gariballa 2006 (7)**	- 69% of patients consumed 0-25% of ONS	- 68% of patients consumed 0-25% of placebo	- Meals: validated food diary<br>- ONS: leftover supplements measured	- Meals: validated food diary<br>- Placebo: leftover supplements measured	NR	NR	NR	NR	Poor	

© 2019 Gomes F et al. *JAMA Network Open.*

	- 6% of patients consumed 26-50%of ONS<br>- 25% of patients consumed 51-75% of ONS<br>- 10% of patients consumed 76-100% of ONS<br>- Meals: NR	- 6% of patients consumed 26-50% of placebo<br>- 22% of patients consumed 51-75%<br>15% 76-100% of placebo<br>- Meals: NR								
**Gazzotti 2003 (8)**	- Meals: NR<br>- ONS: 88% of total ONS offered consumed	Meals: NR	Consumption of each portion of ONS and regular meals measured by direct observation and recorded as all, three quarters, half, one-quarter or none of the portion	Consumption of regular meals measured by direct observation and recorded as all, three quarters, half, one-quarter or none of the portion	NR	NR	Medical reasons rather than patients finding the supplements unpalatable	NR	Good	
**Hickson 2004 (9)**	NR	NR	Weighing the main meals and food records for breakfast, snacks and drinks	Weighing the main meals and food records for breakfast, snacks and drinks	NR	NR	NR	NR	Poor	Data revealed only a “trend towards higher intake in intervention group”
**Hogarth 1996 (10)**	Active energy group (active and placebo vitamin): 31% patients consuming > 50% of offered drinks	Placebo energy group (active and placebo vitamin): 31% patients consuming > 50% of offered drinks	- Measure of unconsumed fluid each day during admission.<br>- Following discharge, estimation of the volume of fluid (in quarters) remaining in each bottle each day.	- Measure of unconsumed fluid each day during admission.<br>- Following discharge, estimation of the volume of fluid (in quarters) remaining in each bottle each day.	NR	NR	Patients were unable to tolerate the large volume (750ml) of sweet, fizzy fluid that was provided.	Patients were unable to tolerate the large volume (750ml) of sweet, fizzy fluid that was provided.	Poor	
**Holyday 2011 (11)**	NR	NR	NR	NR	NR	NR	NR	NR	Poor	
**Huynh 2015 (12)**	- ONS: 90% of compliance.<br>- 85.9% attended at least two sessions of dietary counselling over the study period	- 86.6% attended at least two sessions of dietary counselling over the study period	Dietary intakes: 24-h food recall administered by site dietitians at baseline and every 4 weeks. Compliance with ONS during hospital stay by direct observation; post-discharge compliance by monitoring the	Dietary intakes: 24-h food recall administered by site dietitians at baseline and every 4 weeks.	NR	NR	NR<br>A number of reasons for explaining the high compliance with ONS was provided.	NR<br>A number of reasons for explaining the high compliance with ONS was provided.	Good	

© 2019 Gomes F et al. *JAMA Network Open.*

			return of empty ONS sachets							
**McEvoy 1982 (13)**	NR	NR	NR	NR	NR	NR	NR	NR	Good	Patients “received 2 units of ONS”, according to significant weight gain good adherence must be assumed
**McWhirter 1996 (14)**	- ONS group: 74% of prescription consumed<br>- Enteral feeding group: 78% of prescription consumed	NR	- Meals: food charts completed by nursing staff<br>- ONS and enteral feeding: documented (method NR).	Food charts completed by nursing staff	- ONS group: 71% patients achieving > 80% energy target<br>- Enteral feeding group: 88% patients achieving > 80% energy target	4% patients achieving >80% energy target	NR	NR	Good	
**Munk 2014 (15)**	- Meals: NR<br>- ONS: consumption measured (method and results NR)	- Meals: NR<br>- ONS: consumption measured (method and results NR)	Meals and ONS: visual assessment of amount consumed and recorded in quartiles (0%, 25%, 50%, 75% and 100%) by nursing staff or patients. Daily dietary recall interviews to ensure and verify the content of patients’ dietary records	- Meals and ONS: visual assessment of amount consumed and recorded in quartiles (0%, 25%, 50%, 75% and 100%) by nursing staff or patients. Daily dietary recall interviews to ensure and verify the content of patients’ dietary records	- 76% of patients achieved >75% energy target.<br>- 66% of patients achieved >75% protein target	- 28% of patients achieved >75% energy target.<br>- 12% of patients achieved >75% protein target	NR	NR	Good	
**Neelemaat 2012 (16)**	- Adherence to oral nutritional support: 80%<br>- Adherence to telephone counselling by dietician: 96%	NR	NR	NR	NR	NR	NR	NR	Good	
**Ollenschläger 1992 (17)**	NR	NR	NR	NR	NR	NR	NR	NR	Good	Severely observed patients and interventions, therefore adherence probably good
**Potter 2001 (18)**	NR	NR	- Percentage of supplement consumed recorded<br>- Weighed dietary intakes of voluntary food, (ie, normal diet and snacks)	Weighed dietary intakes of voluntary food, (ie, normal diet and snacks)	NR	NR	Reduced oral intake for medical reasons rather than patients finding the supplements unpalatable.	NR	Good	Observed ONS intake, compliance was good, 50% of patients consumed a mean additional intake of 430 to 540 kcal/d, and a further 25% of patients consumed a

© 2019 Gomes F et al. *JAMA Network Open.*

										mean additional intake of at least 270 kcal/d
**Roberts 2003 (19)**	- 52% consumed 80–100% of total sip-feed provided<br>- 22% consumed 79–50%<br>- 26% consumed less than 50%<br>- Meals: NR	NR	- Counting of the number of sip feed consumed throughout hospital stay<br>- Meal: NR	NR	48% with total energy intake ≥ minimum energy requirements	25% with total energy intake ≥ minimum energy requirements	The reason for non-compliance was generally medical, e.g. no oral intake, vomiting	NR	Good	
**Rüfenacht 2010 (20)**	NR	NR	Meals weighed before and after consumption	Meals weighed before and after consumption	- 94% of patients achieving >75% energy requirements<br>- 78% of patients achieving >75% protein requirements	- 61% of patients achieving >75% energy requirements<br>- 67% of patients achieving >75% protein requirements	NR	NR	Good	
**Ryan 2004 (21)**	NR	NR	Weighing of food and drinks before and after meals	Weighing of food and drinks before and after meals and adding nutrients provided by ONS (method for assessing ONS consumption NR)	NR	NR	NR	NR	Good	Energy intake was assessed over 3 consecutive days, intervention group consumed 800 kJ more/d
**Saudny-Unterberger 1997 (22)**	NR	NR	Food and ONS: calorie count verified by 24-h dietary recall	Food: calorie count verified by 24-h dietary recall	NR	NR	NR	NR	Good	Intervention group consumed significantly more energy/ kg body weight (39 kcal/kg/d) than did the control group
**Schuetz 2019 (23)**	NR	NR	Nutritional intake reassessed every 24–48 h throughout the hospital stay by the trained registered dietician based on daily food records for each patients	Nutritional intake reassessed every 24–48 h throughout the hospital stay by the trained registered dietician based on daily food records for each patients	78% reached both nutritional goals (energy and protein) during the first 10 days	48% reached both nutritional goals (energy and protein) during the first 10 days	Patient, treatment, and hospital factors (e.g., delay or refusal to start enteral or parenteral nutrition by the patient, early discharge of patients, diagnostic exams interfering with nutritional support) may have prevented full adherence to the protocol	NR	Good	The daily re-assessment by dietitians and individualized nutritional care plan is probably contributing to a good adherence in the intervention group
**Somanchi 2011 (24)**	NR	NR	NR	NR	NR	NR	NR	NR	Poor	

© 2019 Gomes F et al. *JAMA Network Open.*

										No information therefore poor adherence assumed
**Starke 2012 (25)**	- Meals: all patients had at least one meal during hospitalisation which was consumed less than 75%<br>- ONS: NR	- Meals: NR<br>- ONS:<br>- 40% of the patients taking less than one quarter<br>- 9% taking 25-50%<br>- 16% taking 50-75%<br>- 35% taking three quarters or more.	- Meals: The consumed part of each food item was visually estimated and recorded.<br>- Snacks, drinks and ONS: reported by ward staff	The consumed part of each food item was visually estimated and recorded	- 83% managed to reach a mean daily energy intake ≥75% threshold of their individual estimated total energy expenditure	- 30% managed to reach a mean daily energy intake ≥75% threshold of their individual estimated total energy expenditure	NR	NR	Good	
**Vermeeren 2004 (26)**	- Meals: NR<br>- ONS: 98% (± 2%) of supplements provided consumed	- Meals: NR<br>- Placebo: NR	- Meals: dietary records and use of an automated food distribution system. Verification by dietician<br>- ONS: monitoring of proportion consumed	- Meals: dietary records and use of an automated food distribution system. Verification by dietician<br>- Placebo: NR	NR	NR	NR	NR	Good	
**Vlaming 2001 (27)**	- Meals: NR<br>- ONS:<br>- 18% consumed <25% of sipfeeds<br>- 19% consumed 25-49.9% of sipfeeds<br>- 29% consumed 50-74.9%<br>- 34% consumed >75%	- Meals: NR<br>- Placebo: NR	Sipfeeds: drug charts	Placebo: NR	NR	NR	NR	NR	Good	
**Volkert 1996 (28)**	- Meals: NR<br>- ONS:<br>- 55% consumed regularly one per day<br>- 45% consumed one every two days or less	- Meals: NR	- Meals: 3-day weighing records<br>- ONS: consumption recorded daily by visits during hospitalization and every week after discharge	- Meals: 3-day weighing records	NR	NR	Anorexia or dislike	NR	Poor	

NR = Not reported; ONS = oral nutrition supplements

© 2019 Gomes F et al. *JAMA Network Open.*

eReferences

1. Bonilla-Palomas JL, Gamez-Lopez AL, Castillo-Dominguez JC, Moreno-Conde M, Lopez Ibanez MC, Alhambra Exposito R, et al. Nutritional Intervention in Malnourished Hospitalized Patients with Heart Failure. Arch Med Res. 2016;47(7):535-40.
2. Broqvis M, Arnqvist H., Dahlström U, Larsson J, Nylander E, Permert J**.** Nutritional assessment and muscle energy metabolism in severe chronic congestive heart failure — effects of long-term dietary supplementation. European Heart Journal. 1994;15:1641-50.
3. Bunout D, Aicardi V, Hirsch S, Petermann M, Kelly M, Silva G, et al. Nutritional support in hospitalized patients with alcoholic liver disease. Eur J Clin Nutr. 1989;43(9):615-21.
4. Cano-Torres EA, Simental-Mendia LE, Morales-Garza LA, Ramos-Delgado JM, Reyes-Gonzalez MM, Sanchez-Nava VM, et al. Impact of Nutritional Intervention on Length of Hospital Stay and Mortality among Hospitalized Patients with Malnutrition: A Clinical Randomized Controlled Trial. J Am Coll Nutr. 2017;36(4):235-9.
5. Deutz NE, Matheson EM, Matarese LE, Luo M, Baggs GE, Nelson JL, et al. Readmission and mortality in malnourished, older, hospitalized adults treated with a specialized oral nutritional supplement: A randomized clinical trial. Clinical Nutrition. 2016;35(1):18-26.
6. Calvey H. DM, Williams R. Controlled tiral of nutritionan supplementation, with and without branched chain amino acid enrichment, in treatment of acute alcoholic hepatitis. Journal of Hepathology. 1985;1:141-51.
7. Gariballa S, Forster S, Walters S, Powers H**.** A randomized, double-blind, placebo-controlled trial of nutritional supplementation during acute illness. Am J Med. 2006;119(8):693-9.
8. Gazzotti C, Arnaud-Battandier F, Parello M, Farine S, Seidel L, Albert A, et al. Prevention of malnutrition in older people during and after hospitalisation: results from a randomised controlled clinical trial. Age Ageing. 2003;32(3):321-5.
9. Hickson M, Bulpitt C, Nunes M, Peters R, Cooke J, Nicholl C, et al. Does additional feeding support provided by health care assistants improve nutritional status and outcome in acutely ill older in-patients?—a randomised control trial. Clinical Nutrition. 2004;23(1):69-77.
10. Hogarth MB, Marshall P, Lovat LB, Palmer AJ, Frost CG, Fletcher AE, et al. Nutritional supplementation in elderly medical in-patients: a double-blind placebo-controlled trial. Age Ageing. 1996;25(6):453-7.
11. Holyday M, Daniells S, Bare M, Caplan GA, Petocz P, Bolin T**.** Malnutrition screening and early nutrition intervention in hospitalised patients in acute aged care: a randomised controlled trial. J Nutr Health Aging. 2012;16(6):562-8.
12. Huynh DT, Devitt AA, Paule CL, Reddy BR, Marathe P, Hegazi RA, et al. Effects of oral nutritional supplementation in the management of malnutrition in hospital and post-hospital discharged patients in India: a randomised, open-label, controlled trial. J Hum Nutr Diet. 2015;28(4):331-43.
13. McEvoy AW, James OF**.** The effect of a dietary supplement (Build-up) on nutritional status in hospitalized elderly patients. Hum Nutr Appl Nutr. 1982;36(5):374-6.
14. Mcwhirter JP PC**.** A Comparison Between Oral and Nasogastric Nutritional Supplements in Malnourished Patients. Nutrition. 1996;12(7-8):502-6.
15. Munk T, Beck AM, Holst M, Rosenbom E, Rasmussen HH, Nielsen MA, et al. Positive effect of protein-supplemented hospital food on protein intake in patients at nutritional risk: a randomised controlled trial. J Hum Nutr Diet. 2014;27(2):122-32.
16. Neelemaat F, Lips P, Bosmans JE, Thijs A, Seidell JC, van Bokhorst-de van der Schueren MAE**.** Short-Term Oral Nutritional Intervention with Protein and Vitamin D Decreases Falls in Malnourished Older Adults. Journal of the American Geriatrics Society. 2012;60(4):691-9.

© 2019 Gomes F et al. *JAMA Network Open.*

17. Ollenschläger G TW, Konkol K, Diehl V, Roth E. Nutritional behaviour and quality of life during oncological polychemotherapy: results of a prospective study on the efficacy of oral nutritional therapy in patients with akute leukemia. European Jounal of Clinical Investigation. 1992;22.
18. Potter JM, Roberts MA, McColl JH, Reilly JJ. Protein energy supplements in unwell elderly patients--a randomized controlled trial. JPEN J Parenter Enteral Nutr. 2001;25(6):323-9.
19. Roberts M, Potter J, McColl J, Reilly J. Can prescription of sip-feed supplements increase energy intake in hospitalised older people with medical problems? British Journal of Nutrition. 2007;90(02):425.
20. Rufenacht U, Ruhlin M, Wegmann M, Imoberdorf R, Ballmer PE. Nutritional counseling improves quality of life and nutrient intake in hospitalized undernourished patients. Nutrition. 2010;26(1):53-60.
21. Ryan M, Salle A, Favreau AM, Simard G, Dumas JF, Malthiery Y, et al. Oral supplements differing in fat and carbohydrate content: effect on the appetite and food intake of undernourished elderly patients. Clin Nutr. 2004;23(4):683-9.
22. Saundy-Unterberger H. MJG, Gray-Donald K. Impact of Nutritional Support on Functional Status During an Acute Exacerbation of Chronic Obstructive Pulmonary Disease. Am J Respir Crit Care Med. 1997;156:794-9.
23. Schuetz P, Fehr R, Baechli V, Geiser M, Gomes F, Kutz A, et al. Individualised nutritional support in medical inpatients at nutritional risk: a randomised clinical trial. The Lancet. 2019;393(10188):2312-21.
24. Somanchi M, Tao X, Mullin GE. The facilitated early enteral and dietary management effectiveness trial in hospitalized patients with malnutrition. JPEN J Parenter Enteral Nutr. 2011;35(2):209-16.
25. Starke J, Schneider H, Alteheld B, Stehle P, Meier R. Short-term individual nutritional care as part of routine clinical setting improves outcome and quality of life in malnourished medical patients. Clin Nutr. 2011;30(2):194-201.
26. Vermeeren MA, Wouters EF, Geraerts-Keeris AJ, Schols AM. Nutritional support in patients with chronic obstructive pulmonary disease during hospitalization for an acute exacerbation; a randomized controlled feasibility trial. Clin Nutr. 2004;23(5):1184-92.
27. Vlaming S, Biehler A, Hennessey EM, Jamieson CP, Chattophadhyay S, Obeid OA, et al. Should the food intake of patients admitted to acute hospital services be routinely supplemented? A randomized placebo controlled trial. Clin Nutr. 2001;20(6):517-26.
28. Volkert D, Hubsch S, Oster P, Schlierf G. Nutritional support and functional status in undernourished geriatric patients during hospitalization and 6-month follow-up. Aging (Milano). 1996;8(6):386-95.
